# An Efficient Indoor Positioning Method Based on Wi-Fi RSS Fingerprint and Classification Algorithm

**DOI:** 10.3390/s21103418

**Published:** 2021-05-14

**Authors:** Balaji Ezhumalai, Moonbae Song, Kwangjin Park

**Affiliations:** 1 Department of Information and Communication Engineering, Wonkwang University, Iksan 570-749, Korea; balajiezhumalai91@gmail.com; 2 Samsung Electronics, Suwon 497-001, Korea; moonbae.song@samsung.com

**Keywords:** indoor positioning, received signal strength (RSS), Wi-Fi fingerprint, SAP similarity, fingerprint clustering, RSS extraction

## Abstract

Wi-Fi received signal strength (RSS) fingerprint-based indoor positioning has been widely used because of its low cost and universality advantages. However, the Wi-Fi RSS is greatly affected by multipath interference in indoor environments, which can cause significant errors in RSS observations. Many methods have been proposed to overcome this issue, including the average method and the error handling method, but these existing methods do not consider the ever-changing dynamics of RSS in indoor environments. In addition, traditional RSS-based clustering algorithms have been proposed in the literature, but they make clusters without considering the nonlinear similarity between reference points (RPs) and the signal distribution in ever-changing indoor environments. Therefore, to improve the positioning accuracy, this paper presents an improved RSS measurement technique (IRSSMT) to minimize the error of RSS observation by using the number of selected RSS and its median values, and the strongest access point (SAP) information-based clustering technique, which groups the RPs using their SAP similarity. The performance of this proposed method is tested by experiments conducted in two different experimental environments. The results reveal that our proposed method can greatly outperform the existing algorithms and improve the positioning accuracy by 89.06% and 67.48%, respectively.

## 1. Introduction

The rapid development of wireless technology leads people to pay more attention to location-based services (LBS). In recent years, indoor LBS has been widely used in airports, subways, shopping malls, and many other indoor environments for location identification, indoor navigation for the users, and the positioning demands of the different groups of people. Therefore, it is important to build an accurate, reliable, and real-time indoor positioning system to effectively satisfy the public demand for indoor positioning. With the rapid development of smartphones, more sensors and intelligent terminals have become excellent tools for indoor positioning [1,2,3].

Nowadays, the global navigation satellite system (GNSS) is well matured and is commonly used in outdoor positioning technology. LBS requirements in outdoor environments were well addressed and positioned in GNSS [4]. However, the GNSS is not applicable for indoor positioning systems due to its signal becoming weaker after penetrating through indoor environments; hence, it fails to produce reliable position information [5]. Different wireless technologies such as Bluetooth (BLE beacon) [6], radio frequency identification (RFID) [7], ultra-wideband (UWB) [8], geomagnetism [9], visible light [10], and Wi-Fi [11] were used in indoor positioning systems. Among the aforementioned technologies, Wi-Fi is the most effective technology used in indoor positioning research areas due to its low power consumption, high precision, and low cost [3]. The universality and common implementation of Wi-Fi technologies do not require additional hardware implementation, as they work with the existing Wi-Fi network installed.

Time of arrival (TOA) [12], time difference of arrival (TDOA) [13], angle of arrival (AOA) [12], and the fingerprinting method [14] are the different methods used in Wi-Fi-based indoor positioning systems. TOA, TDOA, and AOA require an angle of information or distance between two points. Even though their calculations are simple, they mainly rely on line-of-sight propagation (LOSP) between the user’s position and the Wi-Fi access points (APs) [15]. Therefore, the fingerprinting method is widely used in indoor positioning due to its low power consumption, high accuracy, low cost, and the fact that it does not require the location of the Wi-Fi APs and the LOSP [16]. In the Wi-Fi fingerprinting method, the signal strength received from the surrounding Wi-Fi APs is used to estimate the user’s position. The features (fingerprints) of a scene are collected and further estimate the user’s location by matching the online measurement of the collected data [17]. It is also known as the RSS-based fingerprinting method, and it can be implemented in any indoor environment where Wi-Fi devices are deployed. The RSS-based fingerprint method has two phases: offline phase (creating radio map) and online phase (estimating user’s location). In the offline phase, the fingerprint database was created by performing a site survey in the indoor area. In the online phase, the RSS is observed from the user’s unknown location and compared with the RSS stored in the fingerprint database through a localization algorithm unit, which estimates the user’s current location.

The fingerprint positioning methods used to estimate the user location in the online phase can be divided into probabilistic and deterministic methods. In the probabilistic method, it is necessary to know the probability distribution of all deducted APs at each RP stored in the fingerprint database. In contrast, the deterministic method does not need to know the statistical probability model of the Wi-Fi RSS. The advantage of this method is generally simple to implement and useful for real-time applications. In the deterministic method, the nearest neighbor-based localization algorithms are most frequently used; there are nearest neighbor (NN), *k*- nearest neighbor (*k* NN), and weighted *k* nearest neighbor (W *k* NN) algorithms. The W *k* NN is the improved classification algorithm of NN and *k* NN, and it is the most popular deterministic localization algorithm used in Wi-Fi RSS fingerprint-based indoor positioning [1,3].

At present, the main research directions for Wi-Fi RSS fingerprint-based indoor positioning are improving the positioning accuracy and reducing the computational complexity. The accuracy of the RSS fingerprint method is far from adequate due to obstacles in the indoor environment, such as fading and shadowing. The online positioning accuracy of Wi-Fi RSS fingerprint-based indoor positioning is fully dependent on the RSS vectors of each RP stored in the offline fingerprint database, so the RSS extraction is really important for constructing a strong and error-free offline fingerprint database. The average value of the collected RSS samples is generally taken as its RP fingerprint value, and it will be stored in the fingerprint database. In [18], an improved location fingerprinting method was proposed using a mean smoothing algorithm to eliminate the gross error in the offline fingerprint database construction. In [19], the average value of a number of selected maximum RSS values is taken as its RP fingerprint value. To avoid the presence of noisy measurements, the data selection method with a closed-form lease square (LS) approach is proposed in [20] to disregard the bad measurements. The localization accuracy can be increased to some extent in RSS-based positioning using particle filtering [21]. However, the average value of RSS used in the above RSS extraction methods cannot accurately reflect the dynamic behavior of Wi-Fi signals. An error handling method for fingerprint collection is proposed in [22], but the RSS extraction process is very laborious and time-consuming compared to other methods.

The large space of indoor environments such as airports and subways has larger fingerprint databases; hence, finding the few nearest neighbors over a large fingerprint database causes high computational overhead that will affect the algorithm’s efficiency. To overcome this issue, clustering-based fingerprinting methods are proposed; instead of searching all the RPs, a particular cluster (which has a group of RPs) will be searched to find the user’s location using the clustering technique. The researchers have proposed different clustering techniques, such as *k*-means [23,24], Bisecting *k*-means [25], smallest enclosing circle [26], and affinity propagation [27,28]. However, the clustering criteria used in the above-mentioned clustering techniques do not consider the nonlinear similarity between RPs, which means the clustering result cannot perfectly reflect the position distribution of RPs. Since the *k*-means method is an unsupervised machine learning algorithm, the data are unlabeled, and the algorithm learns by itself using the dataset. The disadvantage of this method is that it randomly places the centroids to make clusters, and we never know how this algorithm sorted the data. The output obtained may not be what the user was expecting due to a data interpretation mismatch. When *k* is too large, the relatively relevant points might be divided into different clusters, and when *k* is too small, the less relevant points are much more likely to be divided into the same cluster. Cluster matching in an online phase will become inaccurate due to the inconsistency of the clustering criteria. A location-based clustering is proposed in [29]. Since the signal is not distributed equally due to the obstacles in an indoor environment, location-based clustering will lead to positioning errors. The combination of position label assisted clustering and signal weighted Euclidean distance is proposed in [30], which outperforms the traditional W *k* NN with Euclidean distance and a *k*–means clustering algorithm; however, it is only effective for positioning with the line-of-sight APs, and it is not suitable for positioning with non-line of sight APs.

On the other hand, researchers have focused on automatic fingerprint database construction methods such as interpolation and crowdsourcing [31]. A network fingerprint approach is proposed in [32] to auto construct the fingerprint database through Wi-Fi APs. The crowdsource-based adaptive area localization to enable area classification for continuously generated data is proposed in [33], which dynamically subdivides the floor plan into areas based on the quality and features of available training data. The authors of [34] built the Wi-Fi-based indoor navigation application that uses an interpolation method to generate artificial Wi-Fi fingerprints, but the interpolation technique hardly outperformed the accuracy achieved using manual site surveys. The automatic site survey may cause inaccuracy in the fingerprint database, so in this research, our fingerprint collection work is done manually for better demonstration.

The ever-changing dynamics of RSS in indoor environments impact the average value of missing APs, and the nonlinear similarity between RPs of cluster construction was not considered in the above methods. This paper proposes an IRSSMT RSS extraction method and SAP information-based clustering algorithm to tackle the above-mentioned problems of Wi-Fi fingerprint-based indoor positioning. The proposed IRSSMT helps make the RSS smoother and construct an effective fingerprint database, and the proposed SAP information-based clustering algorithm groups the RPs accurately according to its SAP similarity criterion. First, the Wi-Fi signals are collected and stored in RPs using IRSSMT, then the RPs are grouped as clusters according to their SAP similarity in the offline phase. Finally, the W *k* NN algorithm is employed in the online phase to estimate the user’s position.

The rest of this paper is organized as follows: In Section 2, an overview of the proposed method is presented, and the proposed algorithms are described in detail. Section 3 consists of the experimental results and the performance evaluation of the proposed method. In Section 4, we present a discussion of the results. Finally, the conclusion and future works are discussed in Section 5.

## 2. The Proposed Wi-Fi Fingerprint Method

In the following section, we introduce our approach to the efficient indoor positioning method. We describe the overview of the proposed Wi-Fi RSS fingerprint method. Subsequently, we introduce the proposed concept of RSS preprocessing using IRSSMT and SAP information-based clustering. Finally, we describe the online localization algorithm used to select delegate clusters and find user locations.

### 2.1. Overview of the Proposed Wi-Fi RSS Fingerprint Positioning Method

**Figure 1:**
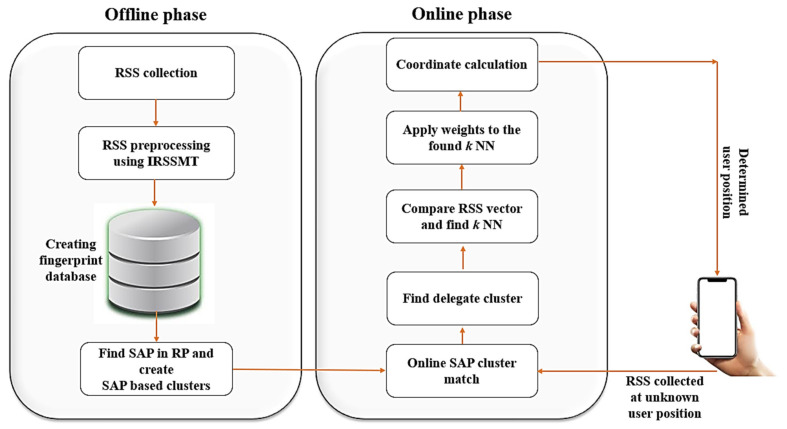
The overall structure of the proposed algorithm.

The overall architecture of the proposed Wi-Fi RSS fingerprint method is shown in Figure 1. Since the proposed method is based on the traditional fingerprint algorithm, it also consists of two phases: the offline phase and the online phase. The traditional preprocessing methods only use the mean value of the RSS samples, and it does not reflect the ever-changing dynamics of RSS and the impact of the average value of its missing APs stored in the fingerprint database; to deal with this problem, we propose an IRSSMT. In the offline phase, we first collect the *N* number of original RSS observation samples at each RP. Then, the collected RSS observations are preprocessed using the median value of the RSS samples to make the RSS smoother and reduce the influence of shadowing and fading. The preprocessed RSS values are then stored in the fingerprint database. The traditional clustering methods cannot position the distribution of RPs; to overcome this problem, we propose an SAP information-based clustering algorithm. We first find the SAP information of RPs, and then that SAP information is assigned as the label of that RPs. Then, using the RP labels as the primary information, the RPs are partitioned into several clusters. This process ensures that the SAP information-based clustering results are consistent with the position distribution of RPs. In the online phase, one particular cluster is chosen as the delegate using SAP cluster matching. The user location is then calculated using the W *k* NN algorithm. The experiment results indicate that the proposed method can greatly improve the positioning accuracy.

### 2.2. Received Signal Strength Preprocessing and Fingerprint Database Construction

**Figure 2:**
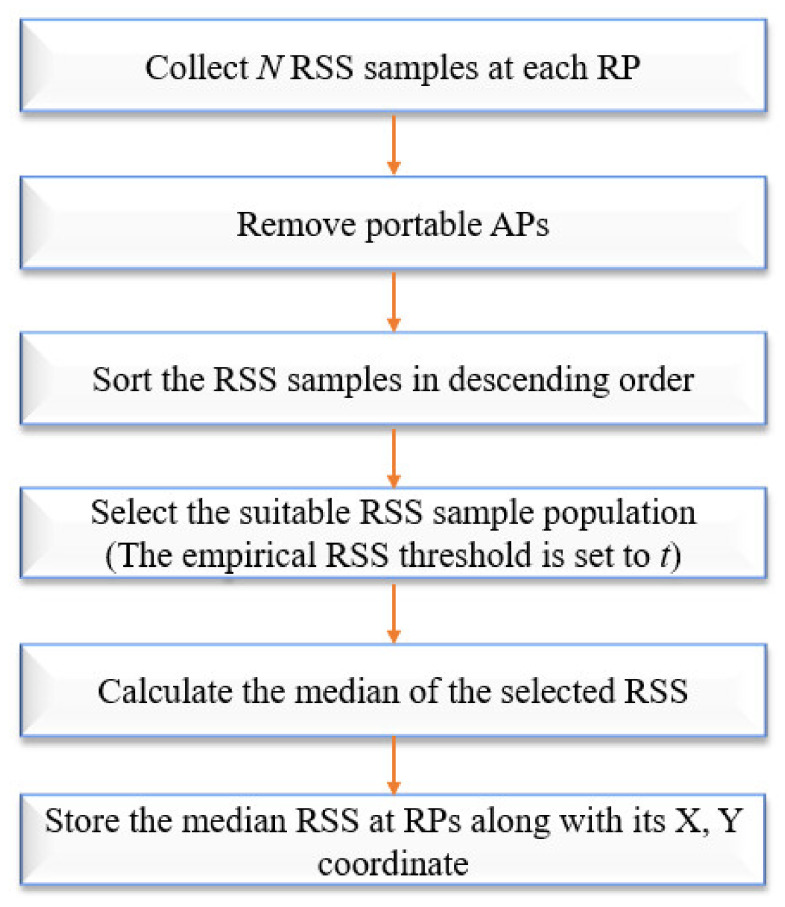
The flow chart of the IRSSMT.

As mentioned earlier, due to the obstacles in indoor environments, Wi-Fi signals can be affected by reflection, diffraction, scattering, and absorption. Therefore, preprocessing the RSS is necessary in order to reduce these influences and make the RSS smoother. The flow chart of the IRSSMT is shown in Figure 2.

In this new and improved method, *N* RSS observations were collected at a 3 s sampling rate at each RP. Any portable APs such as personal Wi-Fi hotspot (WiBro) and temporary Wi-Fi devices placed inside the room for personal use will affect the fingerprint database; to overcome this problem, the portable APs are filtered. Then the RSS samples are sorted in descending order. A weak Wi-Fi signal received from distant APs may affect the positioning accuracy by presenting less stability and reliability, this should be considered during data collection, so the empirical RSS threshold is set to t for rejection of APs with ineffective RSS values. In this research, −90 dBm (*t* = −90 dBm) is set as an empirical threshold value; it is suitable for our scenario, and it may change in other scenarios with different hardware and AP positions. The existing methods, such as the average method, error handling method, and particle filters, may not be able to handle the complex Wi-Fi RSS measurement noise in dynamic and ever-changing indoor environments [19]. For example, according to the distance between RP and AP in the environment, some APs may not be heard in some RPs due to fading and shadowing. In such cases, the missing APs were filled with −99 dBm, and it is considered as the maximum RSS received at the missing APs. These missing AP’s maximum RSS values make a huge difference in the mean value of the collected samples. Therefore, the mean value of the collected RSS and existing methods may not reflect the dynamic behavior of the RSS and the impact of the missing APs. In this case, the median value of the processed RSS samples is a more appropriate representative of RP (see Section 3.1 for a thorough explanation); so, the median value of the original RSS samples is calculated.

The processed RSS vector is stored in the RP and is expressed as:

(1) $$RP_{i}=(RSS_{i1},\;RSS_{i2},\ldots ,\;RSS_{im})$$

where $RP_{i}$ is the processed RSS values stored at the *i*th RP, and *RSS_ij_* is the processed RSS of the *j*th AP at *i*th RP, *i* = 1, 2, …, n, where n is the number of RPs and *j* = 1, 2, …, *m*, where *m* is the number of existing APs.

The RPs are then stored in the fingerprint database along with their RP position (X, Y coordinates), and the format of the fingerprint database is shown in Table 1.

The first column represents the number of RPs in the database, X and Y are the coordinates (RP position) of the *i*th RP measured in the two-dimensional coordinate system of the floor plan. As mentioned in Equation (1), *RSS_ij_* represents the processed RSS value of the *j*th AP at the *i*th RP.

### 2.3. Strongest Access Point Information-Based Clustering Algorithm

We analyzed the similarities between RPs in the offline fingerprint database based on real-time data and found that the set of RPs obtained the strongest RSS value from the particular AP. We continued to analyze the similarities and collected 12 RPs in different locations in a two-dimensional coordinate system and analyzed their RSS values as shown in Table 2.

The strongest RSS value obtained in RP1, RP2, RP3, and RP4 is from AP1, and RP5 to RP8 is from AP2, and RP9 to RP12 is from AP3. According to this real-time result of preprocessed data, we can see that the set of RPs belongs to the particular AP coverage area. Likewise, we can assume that the user is also present somewhere near that same AP coverage area, and those APs are called SAPs.

Inspired by this distribution characteristic of the Wi-Fi signals, we propose an SAP information-based clustering algorithm. The proposed SAP cluster strategy takes more consideration of the signal propagation of the APs and their environment. The AP that obtained the strongest RSS value in $RP_{i}$ will be set as its SAP label and is denoted by:

(2) $$SAP_{i}=argmax_{j=1}^{m}\;RSS_{ij}$$

where $SAP_{i}$ is the SAP label of the *ith* RP, $\;argmax_{j=1}^{m}\;RSS_{ij}$ is the maximum value of RSS obtained from the *j*th AP at the *i*th RP, *m* is the number of APs for *RP_i_* The SAP number will be updated at the SAP label column as shown in Table 2.

In the fingerprint clustering algorithm, the similarity criteria can be calculated using the signal distance (SD) between RPs using Euclidean distance [35], whereas, in this proposed method, the similarity of the SAP labels between RPs is calculated using the following expression:

(3) $$S_{d}\left(RP_{i},\;RP_{j}\right)=\left|SAP_{i}-SAP_{j}\right|$$

where *S_d_(RP_i_, RP_j_)* represents the similarity distance between the SAP label of the *i*th RP and *j*th RP. To obtain the clusters, the SAP information-based clustering is implemented based on the SAP label distance between RPs, as indicated in Equation (3).

The set of clusters are denoted as:

(4) $$C_{l}=\left\{RP_{l1},RP_{l2},\ldots ,RP_{ln}\right\}$$

where *C_l_* is the group of RPs stored at the *l*th cluster, *l* = 1, 2, …, p where *p* is the number of clusters. In this method, the number of clusters is fixed, and it depends on the number of deducted SAPs in the indoor environment. Then, the mean value of the RPs in *C_l_* will be calculated and assigned as the cluster centroid.

The set of cluster centroids are denoted as:

(5) $$CC_{r}=\left\{CC_{1},\;CC_{2},\ldots ,CC_{q}\right\}$$

where *CC_r_* is the number of cluster centroids stored in the *r*th *CC* and *r* = 1, 2, …, *q* where *q* is the number of *CC*.

### 2.4. Online Localization Algorithm

In the online phase, we first collect the RSS vectors at the test point (*TP*), and it is expressed as:

(6) $$TP=\left(RSS_{1},RSS_{2},\ldots ,RSS_{m}\right)$$

where *TP* is the set of RSS vectors collected at the *TP* (user’s position), and *RSS_j_* is the collected RSS of *j*th AP at the *TP*.

#### 2.4.1. Cluster Matching

The main goal of the delegate cluster selection step is to reduce the RP space, through the selection of a delegate cluster centroid that matches the online reading, according to the SAP similarity metric. Instead of comparing the *TP* with all the RPs in the fingerprint database, we first compare the *TP* with the SAP cluster centroids *CC_r_*, then select the particular cluster with the minimum distance as the position set the *TP* belongs to. The delegate cluster is selected using the following equation:

(7) $$D\left(TP,\;CC_{r}\right)=\sqrt{\overset{q}{\underset{r=1}{\sum }}{\left(RSS_{j}-RSS_{rj}\right)}^{2}}$$

where *D(TP, CC_r_)* is the distance between the *TP* and cluster centroids, *RSS_rj_* is the mean RSS value of the *j*th AP at *r*th *CC*. After selecting the delegate cluster using Equation (7), the search space for the algorithm is reduced.

#### 2.4.2. Weighted k-Nearest Neighbor Algorithm

Once the delegate cluster is chosen, the *TP* is compared with all the RPs belonging to that cluster to determine the position of the user by applying the W *k* NN algorithm. The W *k* NN algorithm is an improved classification algorithm of *k* NN, and the working mechanism is to find *k* nearest RPs, which have a minimum signal distance from the RSS observed at the user location, and weighted average the positions of the selected *k* nearest RPs according to their signal distance. The W *k* NN global method will apply weights to all the data points in the database, which also increases the computational overhead. To overcome this issue, the W *k* NN local method is applied in this research. The weights are applied only to the *k* found neighbors. It chooses the RP that has the highest weight and minimum distance to *TP* in the signal space [36].

The *k*-nearest neighbor is calculated by:

(8) $$D\left(TP,\;C_{l}\right)=\sqrt{\overset{n}{\underset{i=1}{\sum }}{\left(RSS_{j}-RSS_{ij}\right)}^{2}}$$

where *D(TP, C_l_)* is the distance between the *TP* and the RPs in the *l*th delegate cluster *C*. The distance values are sorted in ascending order, and the set of *k* nearest RPs can be selected according to their minimum distance. In the *k* NN algorithm, the *k* value can be chosen as:

(9) $$k=\left\{k_{1},\;k_{2},\;\ldots ,\;k_{u}\right\}$$

The coordinate of the *k* nearest RPs with a minimum distance in all *D(TP, C_l_)* is calculated by:

(10) $$\left(x,y\right)=\overset{k}{\underset{i=1}{\sum }}ῶi\left(xi,yi\right)$$

where *(x, y)* is the user position in the Cartesian coordinates, *(xi, yi)* is the position of the *i*th RP, and *ῶi* is the weighting factor of the *i*th *k* selected RP. The weight *ῶi* can be calculated by:

(11) $$ῶi=\frac{\frac{1}{D{\left(TP,\;C_{l}\right)}_{i}^{2}}}{\sum_{j=1}^{k}\frac{1}{D{\left(TP,\;C_{l}\right)}_{j}^{2}}}$$

The closest RP with the highest weight and the lowest distance among the *k* selected RP is considered as the user position.

## 3. Performance Evaluation

**Figure 3:**
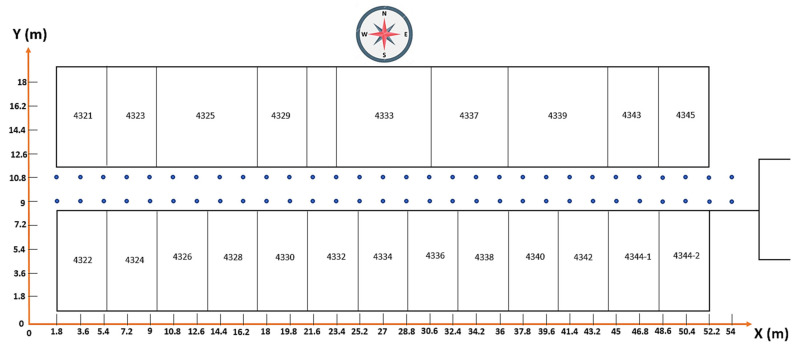
Experimental environment 1. The blue dots represent the RPs, and the total size of the experimental area is 20 m × 60 m.

To validate the performance of our proposed method, two experiments were conducted. Experiment 1 was conducted in the information and communication engineering (ICE) department corridor, 4th floor of the engineering building in Wonkwang University, Republic of Korea. Figure 3 shows the schematic diagram of the corridor on the fourth floor. There were more than 20 APs deployed, and a total of 60 RPs were marked in the experimental field. The accuracy of the position depends on the density of RPs, so the distance between adjacent RPs was set to 1.8 m, and the length of the corridor was 60 m. The position of the RP was measured in the two-dimensional coordinate system and was marked in the X, Y coordinate of the floor plan. A Toshiba Satellite C50-A with an Intel Core i5-3230M CPU 2.60 GHz, 4 GB RAM, and a 64-bit OS was used as the server and the client machine. To deduct APs and collect Wi-Fi RSS data, we used a Wi-Fi network scanner application called “inSSIDer” version 4.4.0 from MetaGeek, LLC. Experiment 2 is explained in Section 3.4.

### 3.1. Results of the Improved RSS Measurement Technique

**Figure 4:**
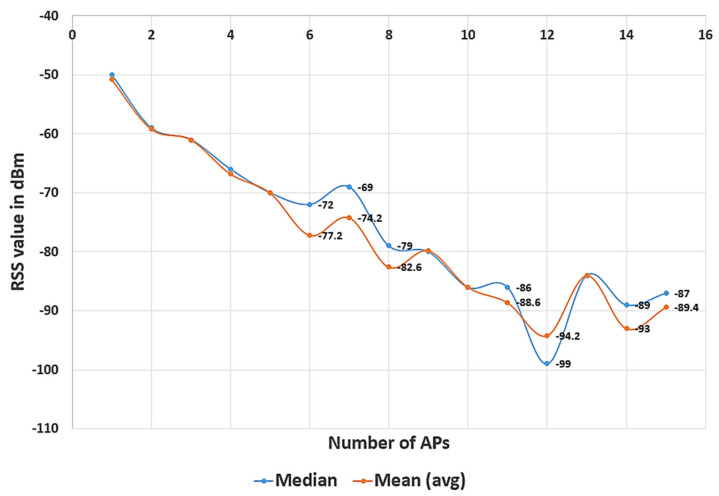
Comparison of mean and median RSS at the RP.

There are more than 15 APs deducted at each RP, but they were filtered as mentioned in Section 2.2. The mean and median value was taken from *N* (*N =* 5) samples of the RP and is compared as shown in Figure 4. The RSS from APs 6, 7, 8, 11, 12, 14, 15 was not heard in some samples due to obstacles in the indoor environment, and these are called missing APs. In this case, −99 dBm was set as its maximum RSS value for those APs as mentioned in Section 2.2, and it makes a huge difference between the mean and median values. Generally, the RSS characteristics are highly based on the mean or probability of the Wi-Fi signal. However, due to fading and shadowing in a complex and ever-changing indoor environment, the characteristics of the RSS are spatially and temporally different. The basic general situations are: (1) The Wi-Fi signal probability distribution is negative-skewed in the presence of weak multipath interference; (2) the Wi-Fi signal probability distribution is positive-skewed if the multipath is stronger than the signal. The mean value of the selected RSS is used in [19]; due to the above-mentioned reasons, the mean value of RSS may not be accurate, and it will affect the positioning accuracy, so the median value of RSS is generally considered to be the most suitable representative of the center of the RSS value.

**Figure 5:**
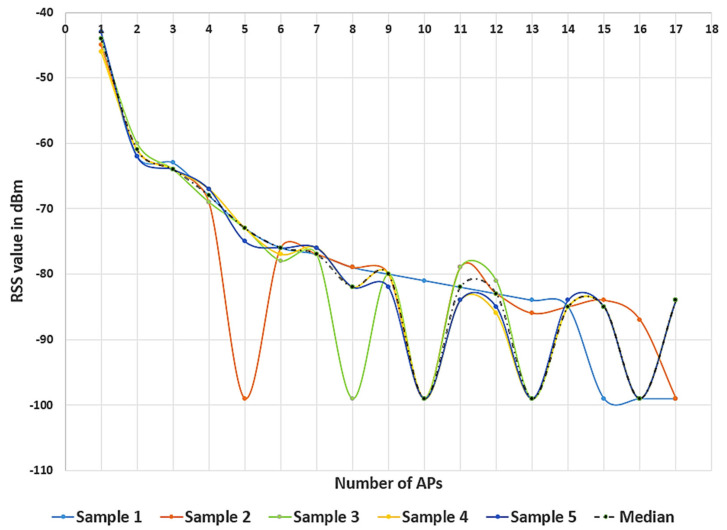
RSS fluctuation of five samples and median RSS value of single RP.

To verify that the improved RSS measurement will reduce Wi-Fi signal fluctuations, we compared the waveforms of the original *N* RSS samples and the processed RSS of the Wi-Fi signal, as shown in Figure 5. There are *N* (*N =* 5) RSS samples collected at the same place from 17 APs, and each sample has a different RSS value from the same AP with a different time interval. This shows the importance of signal preprocessing.

### 3.2. Results of the SAP Cluster Experiment

**Figure 6:**
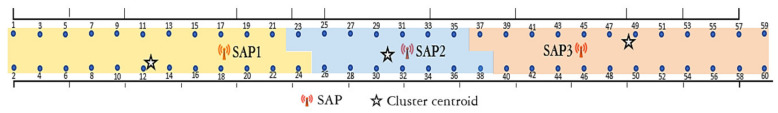
Floor plan with SAP clustered RPs.

The RPs are grouped into three clusters using their SAP information, and their cluster centroid was calculated according to the description in Section 2.3, and as shown in Figure 6. The three different colors represent three different clusters.

**Figure 7:**
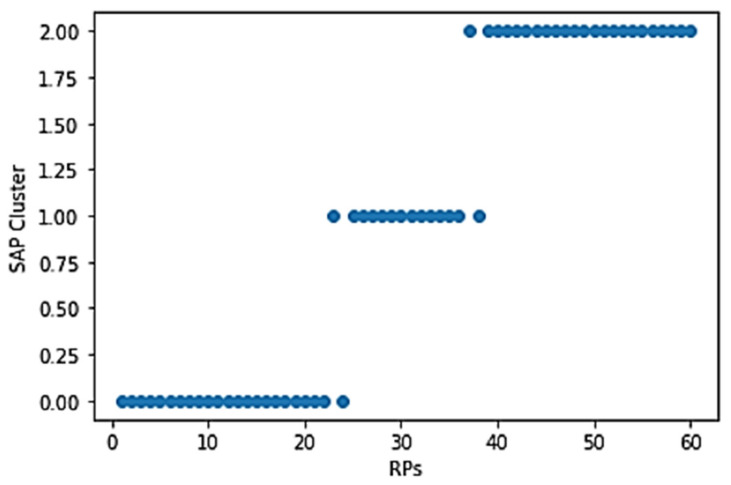
SAP clustered RPs. A group of RPs in y-axis 0, 1, 2 represents cluster 1, 2, 3, respectively.

To compare the performance of the SAP cluster with the traditional *k*-means, we set the *k*-means as a comparison group because it is one of the most widely used robust clustering algorithms for Wi-Fi RSS fingerprint-based indoor positioning [37]. We can see from Figure 6 that the similarity between RPs in cluster 0 shows that the strongest RSS is received from SAP 1, and in clusters 1 and 2, the strongest RSS is received from SAPs 2 and 3, respectively. The SAP cluster is plotted as shown in Figure 7.

Usually, in *k*-means cluster analysis, the Elbow method is used in determining the optimal *k* value [22]. In this research, we used two different *k* values for the *k*-means algorithm, and their result was compared with the proposed SAP algorithm to show the similarity correlation between RPs.

**Figure 8:**
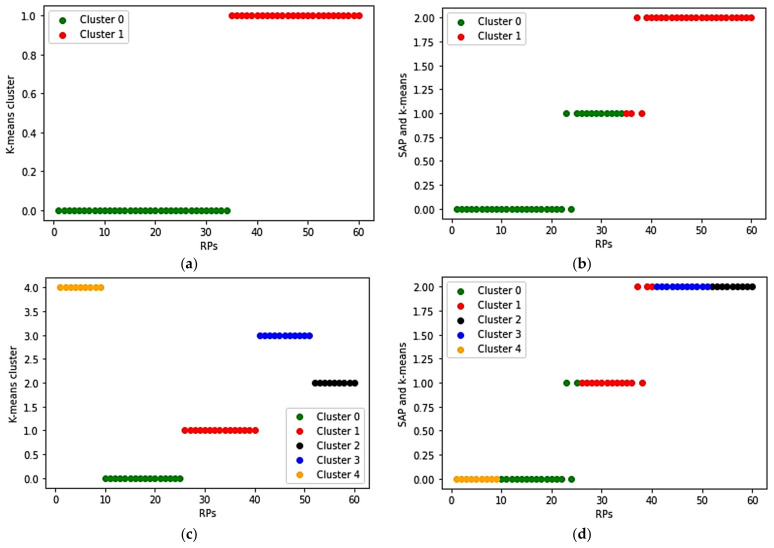
Comparison of *k*-means and SAP clusters: (**a**) *k*-means cluster (*k* = 2); (**b**) *k*-means (*k* = 2) compared with SAP; (**c**) *k*-means cluster (*k* = 5); (**d**) *k*-means (*k* = 5) compared with SAP.

The cluster results of *k*-means and the comparison between *k*-means and SAP are shown in Figure 8. In the *k*-means cluster, *k* = 2, and *k* = 5 are applied and are plotted as shown in (a, c), and (b, d) shows the comparison between the *k*-means and the SAP cluster results. In (b, d), three different groups of RPs on the Y-axis 0, 1, 2 represent three different SAP clusters (cluster 0, 1, 2). The RPs with green and red colors in (b) represent *k*-means clusters where *k* = 2. Likewise, five different colors in (d) represent *k*-means clusters where *k* = 5. The simulation result of the *k*-means algorithm shows that the RPs that are less relevant are divided into the same cluster when the *k* value is small, and the RPs that are relatively relevant are divided into different clusters when the *k* value is large. Whereas, in this proposed SAP cluster, the grouped RPs are more relevant to each other, and the number of clusters is fixed.

### 3.3. Results of the Positioning Experiment

**Figure 9:**
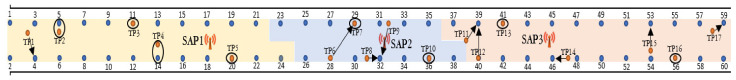
Determined user location of the proposed method. The blue dots represent the RPs, and the orange dots represent the TPs. The RPs pointed with an arrow and circle is the determined user location.

The TPs were taken at the corridor of the experiment field, and the location of the user is estimated using the traditional methods and proposed method. The results of the positioning experiment of the proposed method are shown in Figure 9. The black arrow marks and circles represent the distance between the *TP* (original user location) and the estimated user location.

The error distance between the real user location (*TP*) and estimated user location is calculated using the below equation:

(12) $$DEE=\left(\frac{1}{T}\right)\overset{T}{\underset{i=1}{\sum }}{\left(x_{test}-x_{est}\right)}^{2}+{\left(y_{test}-y_{est}\right)}^{2}$$

where *DEE* is the distance estimation error between the *TP* and the estimated user location, *x_test_, y_test_* is the *X Y* coordinate of the *TP*, and *x_est_, y_est_* is the *X Y* coordinate of the estimated user location.

**Figure 10:**
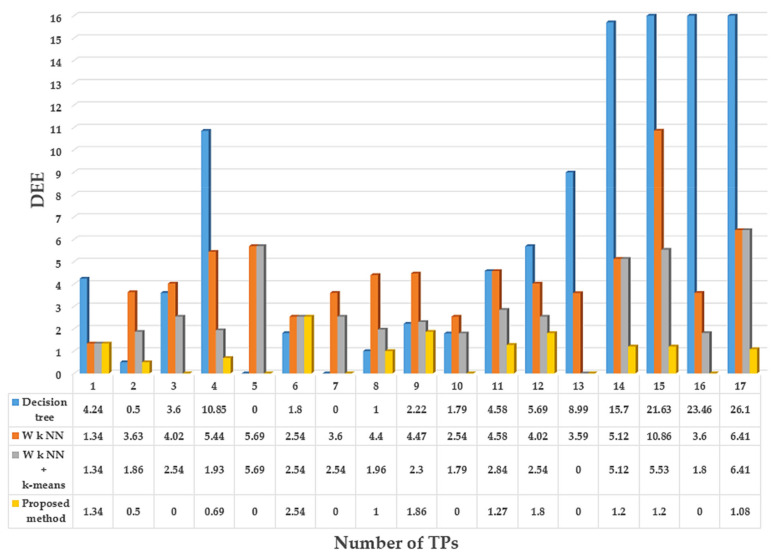
Distance estimation error comparison in meters. Estimated user location of traditional methods and the proposed method.

To evaluate the performance of our proposed algorithm, the positioning accuracy was compared with the traditional W *k* NN algorithm, W *k* NN + *k*-means algorithm, and decision tree algorithm, [25,30] shows that these algorithms are reasonable benchmarks. The comparison of DEE between the proposed algorithm and the other algorithms is shown in Figure 10.

As shown in Figure 10, 35% of TPs (6 out of 17 TPs) have achieved the minimum error (0 m) in the proposed method, whereas the W *k* NN, W *k* NN + *k*-means, and decision tree algorithms achieved 0% (0 out of 17 TPs), 5% (1 out of 17 TPs), and 11% (2 out of 17 TPs) respectively. The maximum error of the proposed method is 2.54 m, whereas the maximum error of W *k* NN, W *k* NN + *k*-means, and decision tree algorithm are 10.86, 6.41, and 26.1 m, respectively. The physical distance between the *TP* and the estimated user location is more consistent with the proposed method.

### 3.4. Experimental Environment 2

**Figure 11:**
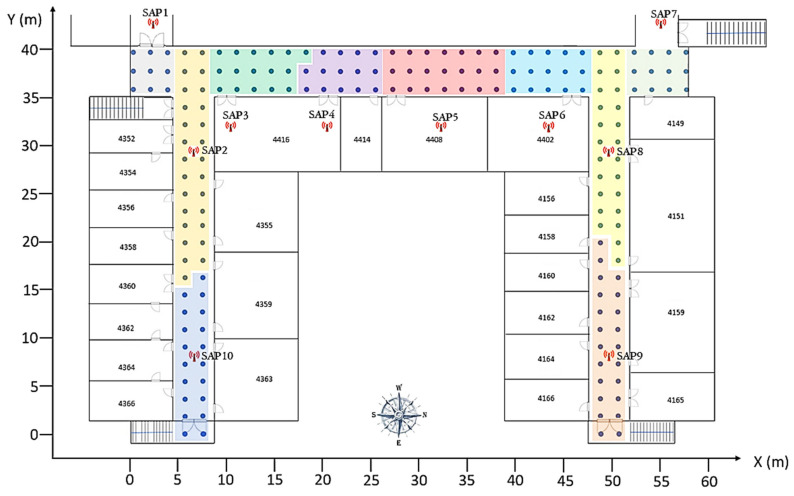
Experimental environment 2. Blue dots represent the RPs, and the size of the experimental field is 60 m × 40 m.

To validate the applicability of our proposed method in a different scenario with different hardware and AP positions, we experimented with our method in a larger indoor environment. Experiment 2 was conducted on the electronics convergence engineering (ECE) department, which is located on the 4th floor of the engineering building. The schematic diagram and the clustering results of experimental environment 2 are shown in Figure 11. A total of 179 RPs were marked in the experimental field, and the distance between adjacent RP was 1.8 m. A total of 895 RSS samples were collected and stored in 179 RPs after preprocessing according to Section 2.2. Overall, 25 APs were deducted during the site survey, and 10 APs were selected as SAPs according to their signal distribution in the environment. The RPs were grouped into 10 different clusters according to their SAP information. Different colors represent different clusters in Figure 11. The TPs were collected at different locations, and the user position is finally estimated.

## 4. Discussion

In this section, we examine the results of the proposed method and various algorithms used to acquire the user location. We collected 17 TPs in experimental environment 1 and 35 TPs in experimental environment 2. We analyzed the distribution of error distance using the cumulative distribution function (CDF), which is a method used to describe the distribution of error distance, and it is predominantly useful to observe the DEE as a cumulative error distribution function. For convenience, the CDF was adopted for the theoretical analysis in this paper. As indicated in [38], it is probable that a normal function variant is between 0 and 1, or it can be explained as the probability at which specific error occurred as in the following equation:

(13) $$CDF\left(d\right)=\frac{2}{\sqrt{\pi }}\overset{d}{\underset{0}{\int }}error^{-t^{2}}dt$$

**Figure 12:**
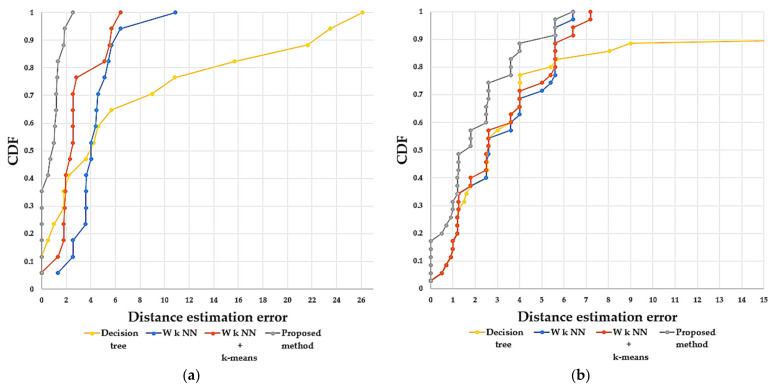
CDF of distance estimation error. (**a**) Experimental environment 1. (**b**) Experimental environment 2. The X-axis represents the error distance in meters, and the Y-axis represents the probability of DEE.

Using the above equation, we could identify the probability of positioning error at some definite distance. The probability of DEE is calculated, and the CDF is shown in Figure 12.

As shown in Figure 12, in experiment environment 1, the probability of getting less than 2 m of DEE in the proposed method is 0.941, whereas in the W *k* NN, W *k* NN + *k*-means, and decision tree are 0.058, 0.411, and 0.352, respectively. In experiment environment 2, the probability of getting less than 2 m of DEE in the proposed method is 0.571, whereas in the W *k* NN, W *k* NN + *k*-means, and decision tree are 0.371, 0.4, and 0.371, respectively. The CDF of the distance estimation error shows that the proposed method obtained a higher probability of better accuracy in both experiment environments 1 and 2 compared to the other methods, which proves that the proposed method is not limited to specific scenarios. The main reason for the improved accuracy is that the SAP cluster divides the RPs more accurately according to its SAP similarity between RPs. In this case, the TPs collected at the boundary of the clusters are well classified into its delegate cluster during simulation, which helps to avoid the error. The better clustering results have produced better positioning accuracy.

### 4.1. Computational Complexity

The main purpose of using clustering algorithms is to reduce the computational complexity of the online localization phase. Furthermore, it will lead to an improvement in positioning accuracy by eliminating possible outliers [35].

The W *k* NN algorithm simply compares the *TP* with the fingerprint database and calculates the distance between *TP* and RPs. Then, the *k* nearest RPs (*k* = 4) is found from the fingerprint database. Finally, the nearest RP with minimum distance and highest weight among the *k* found neighbors is considered to be the user location. In this algorithm, to find the user location, the *TP* is compared with all the RPs that are stored in the fingerprint database, which causes high computational complexity.

The W *k* NN + *k*-means algorithm first groups the RPs and makes clusters. Instead of comparing the *TP* with all the RPs in the database, only the particular cluster with minimum distance to the *TP* is taken for the next process. Finally, the W *k* NN algorithm is performed in that particular cluster to find the user location. For example, if there are 1000 RPs in the fingerprint database, it will be divided into several clusters using the *k*-means algorithm (if *k* = 10, each cluster will have 100 RPs), instead of comparing the *TP* with all the 1000 RPs in the fingerprint database, it selects the single suitable cluster and calculates the user position by comparing only 100 RPs in that selected cluster. The remaining 900 RPs will be omitted, which helps to reduce the computational complexity. Meanwhile, as mentioned in Section 1, the *k*-means clustering algorithm randomly places its centroids to the fingerprint database to make clusters, and it does not consider the nonlinear similarity between RPs, which seriously reduced the positioning accuracy during the simulation.

Whereas, in this proposed method, the clusters are made perfectly by grouping the RPs using its SAP similarity. Therefore, the accuracy of this method is better than the other two compared algorithms. Since the proposed method has a clustering technique, so it can also reduce the computational complexity.

The Big O notation is used in this paper for complexity analysis. It is the most common metric for calculating runtime complexity (execution time). It describes the execution time of a task concerning the number of operations required to complete it. All these traditional and proposed methods are based on the W *k* NN algorithm and the prediction time complexity of W *k* NN is expressed by:

(14) $$O\left(k\times n\times d\right)$$

where *k* is the number of neighbors that we considered in the algorithm (*k* = 4), *n* is the number of RPs in the training dataset, and *d* is the data dimensionality (RSS vectors received from different APs). Equation (14) loop through all points *k* times. First, it computes the distance between *TP* and RPs in the training dataset, then it remembers the index of the element with the smallest distance and adds the class at found RP to the counter. Finally, it returns the class with the most votes as a determined user location. When the number of searching points (RP) in the dataset is high, the prediction time complexity will be higher. The dataset and the execution time are shown in Table 3.

As shown in the above table, there are no clusters in the W *k* NN method, and the *TP* is directly compared with all the RPs in the fingerprint database. In contrast, the W *k* NN + *k*-means algorithm has three clusters in both experimental environments 1 and 2 (the number of clusters in *k*-means comes from the Elbow method where *k* = 3). On the other hand, the proposed method has three clusters in experimental environment 1, and 10 clusters in experimental environment 2 (the number of clusters in the proposed method comes from the SAP cluster technique). It can be seen from the results that the execution time highly depends on the number of RPs stored in that cluster, and the user location calculation is significantly reduced in the proposed method.

### 4.2. Error Statistics

To show the efficiency of the proposed method more intuitively, Table 4 lists the comparison of error statistics between the proposed method and the other traditional methods.

The error metrics used in Table 4 are followed from [30]. As we can see from the above table, the 50% and 75% mean error of DEE of the proposed method is less than the other compared methods in both experimental environments. The mean error of DEE is used in this paper as a metric to judge the positioning accuracy. The mean error of the proposed method in experimental environment 1 is 80.94%, 69.69%, and 89.06%, and in experimental environment 2 is 32.80%, 32.58%, and 67.48% lower than the mean error of the other methods used for comparison. We can see from Figure 10 and Figure 12 and Table 3 and Table 4 that the proposed indoor positioning algorithm outperforms traditional W *k* NN, W *k* NN + *k*-means, and decision tree indoor positioning algorithms. The results indicate that the proposed method can significantly improve positioning accuracy.

The positioning accuracy of this Wi-Fi RSS fingerprint-based indoor positioning highly relies on the number of acquired RPs and RSS observed in the indoor environment. Meanwhile, the ever-changing APs in the indoor environment (for example, the addition of new APs and removal of damaged existing APs) are one of the nagging issues of this method. Moreover, the changes in the indoor infrastructure (modifying the structure of the environment, for example, adding new rooms and removing existing partitions) will also reflect the fingerprint database. Due to the above-mentioned reasons, the positioning accuracy of this method is limited.

## 5. Conclusions

In this paper, we presented an efficient Wi-Fi indoor positioning method based on the IRSSMT Wi-Fi signal extraction method and the SAP information-based clustering algorithm. The proposed method is mainly intended to cope with the issue of multipath interference in indoor environments, impacts of the average value of missing APs, and randomly constructed clusters with randomly placed cluster centroids. The proposed method is based on the analysis of the characteristics of the Wi-Fi signals. The selected RSS median value is employed for better offline fingerprint database construction, and the SAP information reflects the better RP similarity and helps to create the most appropriate cluster. The performance of this proposed method is evaluated by experiments in two different indoor environments. The results show that the proposed IRSSMT and SAP information-based clustering algorithm outperform the traditional indoor positioning algorithms. Besides, the SAP information-based clustering algorithm produces significantly better clustering solutions quite consistently according to the SAP similarity measures of cluster quality, and the proposed algorithm has better-improved accuracy by 89.06% and 67.48% when compared to the other two algorithms. In addition, the run time of the proposed method is incredibly attractive, which greatly reduced the computational complexity. For future work, we will analyze and modify various filters, clustering algorithms, and localization algorithms to enhance the environmental adaptability of fingerprint positioning.

## Figures and Tables

**Table 1:** Fingerprint database format.

RP_s_	X	Y	RSS of AP_1_	RSS of AP_2_	RSS of AP_3_	…	RSS of AP_m_
1	X_1_	Y_1_	RSS_11_	RSS_12_	RSS_13_	…	RSS_1m_
2	X_2_	Y_2_	RSS_21_	RSS_22_	RSS_23_	…	RSS_2m_
3	X_3_	Y_3_	RSS_31_	RSS_32_	RSS_33_	…	RSS_3m_
…	…	…	…	…	…	…	…
n	X_N_	Y_N_	RSS_n1_	RSS_n2_	RSS_n3_	…	RSS_nm_

**Table 2:** Analysis of similarities between RPs.

RPs	RSS of AP1	RSS of AP2	RSS of AP3	RSS of AP4	RSS of AP5	RSS of AP6	SAP Label
1	−48	−62	−71	−55	−72	−81	1
2	−48	−65	−68	−60	−76	−78	1
3	−48	−59	−71	−56	−69	−74	1
4	−49	−60	−70	−60	−78	−78	1
5	−62	−38	−59	−78	−57	−63	2
6	−64	−35	−61	−75	−61	−66	2
7	−72	−48	−56	−84	−70	−60	2
8	−67	−43	−59	−77	−67	−61	2
9	−76	−70	−57	−99	−80	−76	3
10	−70	−66	−48	−99	−86	−69	3
11	−77	−66	−54	−90	−84	−77	3
12	−74	−70	−54	−90	−84	−78	3

**Table 3:** Structure of dataset and execution time based on simulation data.

Method	Experimental Environment 1			Experimental Environment 2		
	Number of Clusters	Number of Rps in Each Cluster	Execution Time	Number of Clusters	Number of Rps in Each Cluster	Execution Time
**W *k* NN**	0	60	0.059000	0	179 RPs	0.300371
**W *k* NN** + ***k*-means**	3	Cluster1–24 RPs	0.028958	3	Cluster1–74 RPs	0.094965
		Cluster2–20 RPs	0.022051		Cluster2–65 RPs	0.061348
		Cluster3–16 RPs	0.026892		Cluster3–40 RPs	0.042885
**Proposed** **method**	3			10	Cluster1–9 RPs	0.014109
					Cluster2–27 RPs	0.015463
					Cluster3–16 RPs	0.016708
		Cluster1–23 RPs	0.014309		Cluster4–14 RPs	0.011992
		Cluster2–14 RPs	0.013577		Cluster5–21 RPs	0.015547
		Cluster3–23 RPs	0.016508		Cluster6–15 RPs	0.019880
					Cluster7–24 RPs	0.013052
					Cluster8–12 RPs	0.015146
					Cluster9–22 RPs	0.014271
					Cluster10–19 RPs	0.015090

**Table 4:** Comparison of error statistics of the traditional algorithms and proposed method.

Method	Experimental Environment 1			Experimental Environment 2		
	50% Error	75% Error	Mean Error	50% Error	75% Error	Mean Error
**W *k* NN**	4.02	4.58	4.46	2.6	5.4	3.14
**W *k* NN** **+** ***k*** **-means**	2.3	2.54	2.86	2.5	5	3.13
**Decision** **tree**	3.6	8.99	7.77	2.54	4.02	6.49
**Proposed method**	0.69	1.2	0.85	1.25	2.6	2.11

## Data Availability

Not applicable.
